# Reviewed article: Almeida ALS, Sousa TMS, Caldeira TCM, Claro RM. Association between adherence to the Food Guide golden rule and health characteristics among adult Brazilian women: a cross-sectional study with VIGITEL data, 2018-2021. Epidemiol Serv Saude. 2025:34;20240232.

**DOI:** 10.1590/S2237-96222025v34e20240232.b

**Published:** 2025-04-11

**Authors:** Maria Cecília Ramos de Carvalho

**Affiliations:** 1 Universidade Federal de Minas Gerais

**Table:** 

Rating	Excellent	Good	Average	Below average	Poor
Interest		✓			
Quality				✓	
Originality			✓		
Overall			✓		

**Table:** 

Questionnaire	Yes	No	Not applicable
Does the manuscript contain new and significant information to justify publication?	✓		
Does the Abstract (Summary) clearly and accurately describe the content of the article?	✓		
Is the problem significant and concisely stated?	✓		
Are the methods described comprehensively?		✓	
Are the interpretations and conclusions justified by the results?		✓	
Is adequate reference made to other work in the field?	✓		

## Manuscript Structure:

Length of article is: Adequate

Number of tables is: Too many

Number of figures is: Adequate

Please state any conflict(s) of interest that you have in relation to the review of this paper (state “none” if this is not applicable): None

## Comments

Inicialmente apresentam-se questões gerais relativas à forma do manuscrito, para que o mesmo possa ser considerado para publicação:

Remover o resumen, o abstract e o quadro de contribuições do estudo, conforme as novas normas de submissão de manuscritos.

Indica-se a necessidade de revisão das normas para a devida adequação do manuscrito à sua versão atualizada

A seguir são apresentadas questões relativas ao conteúdo do manuscrito:

Quanto ao resumo:

Sugere-se inverter a ordem de explicação da associação encontrada: doenças crônicas e autoavaliação negativa da saúde se associaram a uma menor chance de adesão à regra de ouro

Questões relativas à introdução:

As duas linhas finais do primeiro parágrafo podem ser suprimidas, pois apenas repetem a regra de ouro do GAPB

Cabe apresentar os outros grupos de alimentos conforme a classificação NOVA, para que um leitor não familiarizado com o tema possa compreendê-la

Apresentam-se questões relativas aos métodos:

Qual foi o motivo para os autores haverem escolhido apenas características de saúde ao avaliar os fatores associados ao desfecho, e terem considerado as características sociodemográficas como variáveis de controle de confusão em vez de fatores também associados ao desfecho? Há evidências de que outras características sociodemográficas dos indivíduos se associam à adesão ou não a recomendações nutricionais, e tais características poderiam ter sido exploradas neste trabalho pois estão disponíveis nos bancos de dados do VIGITEL

Caso haja disponibilidade de dados de raça/cor da pele dentre as informações coletadas pelo VIGITEL nos anos avaliados, é de fundamental importância incluí-los nas análises, visto a sua interação com outras características sociodemográficas na produção de desfechos em alimentação, nutrição e saúde

O escore de adesão à regra de ouro do GAPB e suas respectivas categorias de classificação foram validados? Se sim, quais componentes de validade foram investigados, e qual(is) instrumento(s) foi(ram) tomado(s) como referência(s) para tal validação? Caso o escore do presente artigo não tenha sido validado, torna-se necessário apresentar uma justificativa para tanto

Considerando que os autores têm acesso aos dados de mais de uma edição do VIGITEL, seria esperado que se analisasse a tendência de adesão à Regra de Ouro do GAPB ao longo dos anos, conjuntamente com os fatores associados à alta adesão. Há possibilidade de inclusão de tal análise no presente artigo, de maneira adicional às análises já realizadas?

A respeito dos resultados:

As informações apresentadas nas tabelas 1 e 2 podem ser consolidadas em uma tabela única, o que tornará a leitura do manuscrito mais fluida

A sigla RO para se referir à regra de ouro não contribui para a fluidez do texto, portanto, recomenda-se que não seja usada

Na transição entre as páginas 12 e 13, não há menção da obesidade como característica associada à alta adesão, o que contrasta com os dados da tabela 3

Quanto à discussão:

No início da discussão, afirma-se que “mulheres com baixa adesão à Regra de Ouro são mais jovens, com nível intermediário de escolaridade e sem companheiro”. Entretanto, na forma atual do manuscrito, as características sociodemográficas foram utilizadas apenas como variáveis de controle de confusão e portanto não deveriam ser discutidas. Por outro lado, reforça-se a relevância de ampliar o objetivo do estudo e as análises realizadas, para se considerar fatores mais diversos que possam se associar à adesão à regra de ouro do GAPB. A primeira sugestão apresentada para a seção de Métodos diz respeito a esta ampliação das análises, e caso seja adotada pelos autores, a afirmativa problematizada da discussão estará de acordo com o objetivo revisado e as análises reinterpretadas

Ainda no primeiro parágrafo da discussão, urge reescrever a frase “A adesão moderada e alta à Regra de Ouro foi associada a um risco reduzido...”, dado que o delineamento transversal não permite a avaliação de risco como medida de força de associação

As duas últimas frases do primeiro parágrafo da discussão também não são adequadas ao delineamento transversal, visto que implicam em uma interpretação de análises de efeito que não cabem no presente estudo

No início da página 15, após afirmar que “as mulheres também desempenham um papel fundamental no planejamento das refeições familiares”, é imprescindível apresentar outra recomendação do GAPB, referente ao compartilhamento de habilidades culinárias e à divisão de tarefas domésticas relacionadas à alimentação, para não ampliar o estereótipo da alimentação como obrigação exclusivamente feminina

Ainda na página 15, a afirmativa “Os resultados do nosso estudo sugerem que a adesão à Regra de Ouro do Guia Alimentar para a População Brasileira não só protege contra DCNT, mas também pode reduzir o risco de mortalidade pela promoção de um maior consumo de alimentos in natura e minimamente processados e diminuição da ingestão de alimentos ultraprocessados” não é corroborada pelos dados e deve ser suprimida, visto que medidas de risco não são avaliáveis em estudos transversais

No final da página 15 há mais uma afirmativa que não é corroborada pelos dados e precisa ser reescrita ou suprimida: “a adesão a um aspecto da regra promove a adesão ao outro”

Na página 16, ao citar iniciativas do Estado para a promoção, proteção e apoio à alimentação adequada e saudável, é imprescindível citar o Programa Nacional de Alimentação Escolar e as modalidades do Programa de Aquisição de Alimentos, tendo em vista a importância de ambos na criação de mercado para produtos da agricultura familiar e possível expansão da oferta de tais produtos para a população em geral. Entretanto, também é indispensável mencionar as fragilidades da regulamentação atual, especialmente no que tange às tentativas de taxação de alimentos ultraprocessados e de regulamentação de sua publicidade

Ao final da página 16 e na página 17, é necessário substituir as ocorrências das palavras “impacto” e “efeito” por outro conceito que não carregue um significado de causalidade (impossível de se avaliar em estudos transversais)

Quanto à conclusão do estudo, recomenda-se a sua revisão conforme os comentários apresentados anteriormente, para que não sejam feitas afirmações excedendo as possibilidades de inferência de um estudo transversal.

